# NPM-hMLF1 fusion protein suppresses defects of a *Drosophila* FTLD model expressing the human *FUS* gene

**DOI:** 10.1038/s41598-018-29716-9

**Published:** 2018-07-26

**Authors:** Itaru Yamamoto, Yumiko Azuma, Yukie Kushimura, Hideki Yoshida, Ikuko Mizuta, Toshiki Mizuno, Morio Ueyama, Yoshitaka Nagai, Takahiko Tokuda, Masamitsu Yamaguchi

**Affiliations:** 1 Department of Applied Biology, Kyoto Institute of Technology, Matsugasaki, Sakyo-ku, Kyoto, 606-8585 Japan; 2 The Center for Advanced Insect Research, Kyoto Institute of Technology, Matsugasaki, Sakyo-ku, Kyoto, 606-8585 Japan; 3 Department of Neurology, Graduate School of Medical Science, Kyoto Prefectural University of Medicine, 465 Kajii-cho, Kamigyo-ku, Kyoto, 602-8566 Japan; 4 Department of Molecular Pathobiology of Brain Diseases, Graduate School of Medical Science, Kyoto Prefectural University of Medicine, 465 Kajii-cho, Kamigyo-ku, Kyoto, 602-8566 Japan; 5 Department of Neurotherapeutics, Osaka University Graduate School of Medicine, 2-2 Yamadaoka, Suita, Osaka, 565-0871 Japan

## Abstract

Fused in sarcoma (FUS) was identified as a component of typical inclusions in frontotemporal lobar degeneration (FTLD) and amyotrophic lateral sclerosis (ALS). In FTLD, both nuclear and cytoplasmic inclusions with wild-type FUS exist, while cytoplasmic inclusions with a mutant-form of FUS occur in many ALS cases. These observations imply that FUS plays a role across these two diseases. In this study, we examined the effect of several proteins including molecular chaperons on the aberrant eye morphology phenotype induced by overexpression of wild-type human *FUS* (*hFUS*) in *Drosophila* eye imaginal discs. By screening, we found that the co-expression of nucleophosmin–human myeloid leukemia factor 1 (NPM*-*hMLF1) fusion protein could suppress the aberrant eye morphology phenotype induced by *hFUS*. The driving of *hFUS* expression at 28 °C down-regulated levels of hFUS and endogenous cabeza, a *Drosophila* homolog of hFUS. The down-regulation was mediated by proteasome dependent degradation. Co-expression of *NPM*-*hMLF*1 suppressed this down-regulation. In addition, co-expression of *NPM*-*hMLF*1 partially rescued pharate adult lethal phenotype induced by *hFUS* in motor neurons. These findings with a *Drosophila* model that mimics FTLD provide clues for the development of novel FTLD therapies.

## Introduction

Frontotemporal lobar degeneration (FTLD) defines a group of neurodegenerative brain diseases characterized by frontal and temporal lobe atrophy^1^. Clinically, the patients show behavioral/personality impairments and/or language problems^1^. Amyotrophic lateral sclerosis (ALS) is a neurodegenerative disorder with the progressive degeneration of both upper motor neurons in the motor cortex and lower motor neurons in the brainstem and spinal cord^2^. ALS patients develop aggressive muscle weakness and ultimately die within 3–5 years without artificial respiration^3^. Although these two diseases are different, it was found that they were present in same individuals or associated within the same family^4–6^. Moreover, previous studies show that about 50 percent of ALS patients exerts slight impairments of cognitive functions and behavior, and that over 15 percent may finally develop frontotemporal dementia^7,8^. Therefore, these two diseases are considered to be a part of spectrum. Neuropathological evidences of FTLD are characterized by protein inclusions^1^. Although about 60 percent of FTLD patients show ubiquitin and TAR DNA-binding protein 43 kDa (TDP-43) positive inclusions, four more subtypes are also identified depending on specific protein component of inclusions (FTLD-tau, FTLD-FET, FTLD-UPS and FTLD-ni)^2,9^. Fused in sarcoma (FUS) is identified as a component of typical inclusions in atypical FTLD with ubiquitin-positive inclusions, neuronal intermediate filament inclusion disease and basophilic inclusion body disease, which are neuropathologically categorized as FTLD-FET^10–12^. Notably, FUS is also identified in ALS^13,14^. FUS belongs to the FET family of DNA/RNA binding proteins and contains several domains^15,16^. FUS is predominantly localized in the nucleus, though it is able to shuttle from the cytoplasm to the nucleus mediated by the nuclear transport receptor, transportin1^17^. FUS is also involved in RNA metabolisms including transcription, pre-mRNA splicing, mRNA transport, post-translational modification and miRNA biogenesis^18^. Recently, increased FUS protein levels in FTLD-brain samples were reported^19^. In addition, it was also shown that four mutations in the 3′ untranslated region (UTR) of *FUS* that were identified in ALS patients caused FUS overexpression, indicating the pathological signature of wild-type FUS overexpression in not only FTLD but also some cases of ALS^19,20^. Consistent with these findings, cell and rodent models harboring wild-type human *FUS* (*hFUS*) were established and they support the conclusion that wild-type FUS accumulation, but not the mutant-form of FUS, is sufficient to cause neurodegeneration and neuronal cell death^21–23^.

Polyglutamine (polyQ) disease is a collectively named neurodegenerative disorder including Huntington’s disease, spinal and bulbar muscular atrophy, dentatorubral-pallidoluysian atrophy and spinocerebellar ataxia^24^. These diseases are characterized by aggressive neurodegeneration, behavioral/physical impairments and defects in cognitive functions^24^. Interestingly, it is reported that FUS is a component of nuclear inclusions in some types of polyQ disease^25–27^.

*Drosophila* has a single FUS homolog, cabeza (caz)^28,29^. Loss of endogenous caz showed eye degeneration, severe reduction of eclosion rate, reduced life span, defects at neuromuscular junctions and locomotive disabilities^30,31^. Transgenic flies carrying mutant or wild-type *hFUS* have been also established and they exert similarly severe eye degeneration, pharate adult lethal phenotype, locomotive disabilities and synapse defects that precede neurodegeneration^31–37^. PolyQ disease model flies expressing long polyQ repeats also exhibit severe eye degeneration^38,39^. Interestingly the eye degeneration phenotype was effectively suppressed by co-expression of various proteins, including molecular chaperones^40,41^.

In the present study, we therefore examined the effect of several proteins including molecular chaperons on the aberrant eye morphology phenotype induced by wild-type *hFUS*. By screening, we have found that the co-expression of nucleophosmin–human myeloid leukemia factor 1 (NPM*-*hMLF1) fusion protein could suppress the aberrant eye morphology phenotype induced by *hFUS*. Fusion protein NPM-hMLF1 is produced by the t(3;5) (q25.1;q34) chromosomal translocation, associating with myelodysplastic syndrome (MDS) and acute myeloid leukemia (AML)^42,43^. NPM is a nucleolar localized non-ribosomal RNA binding phosphoprotein that functions as a shuttle protein by transporting ribosomal ribonucleoproteins between the nucleus and the cytoplasm during the assembly of ribosomes^44,45^. hMLF1 protein mainly localizes in the cytoplasm, while its fused form with NPM mainly localizes in the nucleus^43^. The driving of *hFUS* expression at 28 °C down-regulated levels of hFUS and endogenous caz. The down-regulation was mediated by proteasome dependent degradation. Co-expression of *NPM*-*hMLF1* suppressed this down-regulation. In addition, co-expression of *NPM*-*hMLF1* partially rescued pharate adult lethal phenotype induced by *hFUS* in motor neurons.

## Results

### Ectopic expression of hFUS in *Drosophila* eye imaginal discs induces aberrant eye morphology in adults

**Figure 1:**
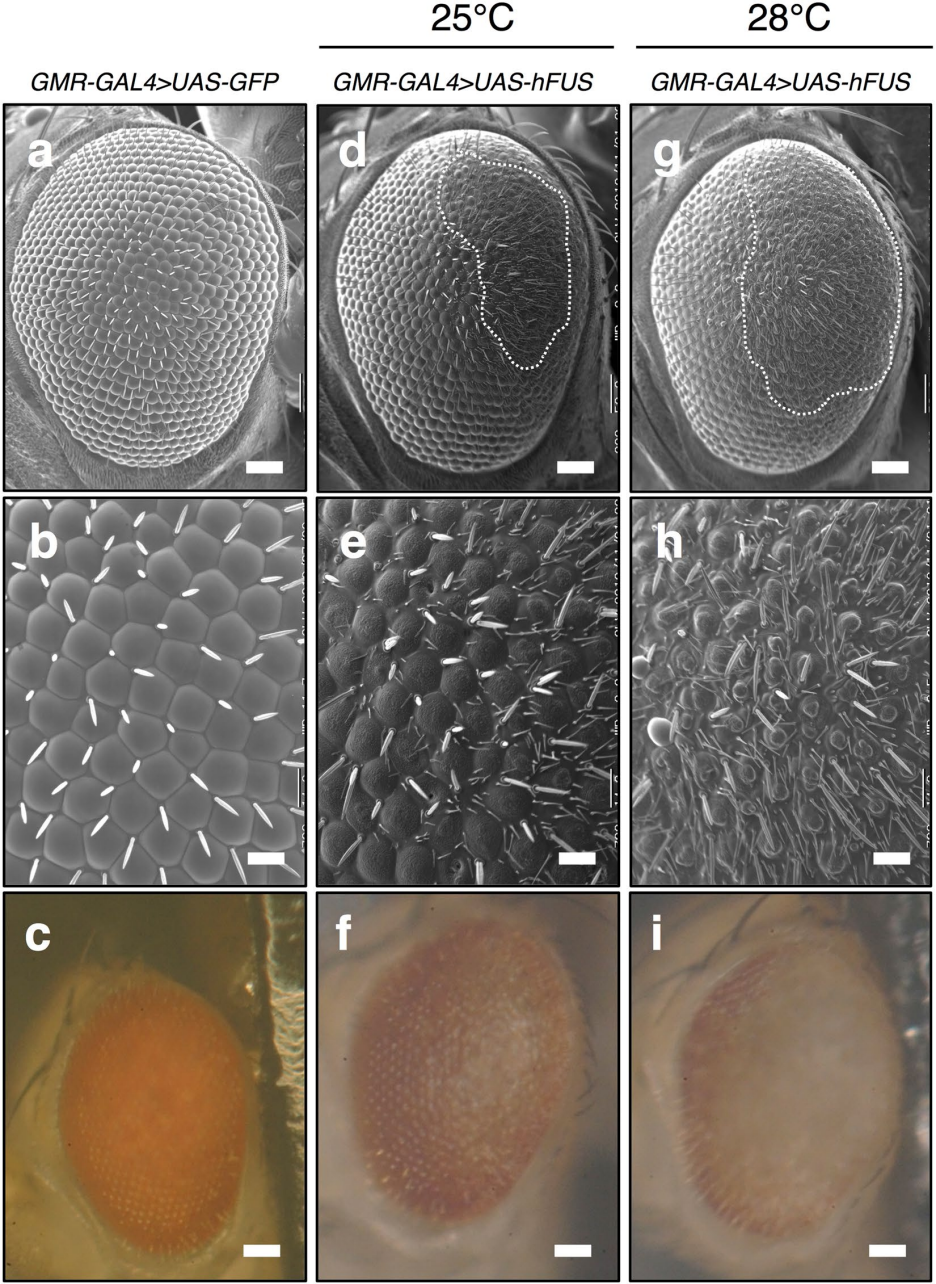
Exogenous expression of *hFUS* in eye imaginal discs exhibited aberrant eye morphology in adults. Scanning (**a**,**b**,**d**,**e**,**g**,**h**) and stereoscopic (**c**,**f**,**i**) micrographs of adult compound eyes. (**a**–**c**) *GMR-GAL4* > *UAS-GFP* (*GMR*-*GAL4*/*Y*; *UAS*-*GFP*/+). (**d**–**i**) *GMR-GAL4* > *UAS-hFUS* (*GMR*-*GAL4*/*Y*; *UAS*-*hFUS*/+). (**b**,**e**,**h**) Enlarged images of (**a**,**d**,**g**) respectively. The eye phenotype of at least five newly eclosed adult male flies of each line was examined and the experiments were done in triplicate. No significant variation in eye phenotype was observed among the five individuals. Anterior is to the left and dorsal to the top. The flies were developed at either 25 °C (**d**–**f**) or 28 °C (**a**,**b**,**c**,**g**,**h**,**i**). The bars indicate 50 μm (**a**,**c**,**d**,**f**,**g**,**i**) and 14.2 μm (**b**,**e**,**h**), respectively. White dotted lines define the area of aberrant eye morphology.

It was reported that ectopic expression of *hFUS* in eye imaginal discs induces aberrant eye morphology^31–33,37^. To confirm the effect of *hFUS* expression on eye morphology, we crossed *w*; *UAS*-*hFUS* /*CyO Act*-*GFP* with *GMR*-*GAL4* driver to express *hFUS* in the region posterior to the morphogenetic furrow (MF) and then inspected the adult compound eyes. Expression of *hFUS* in eye imaginal discs exhibited aberrant eye morphology at 25 °C (Fig. 1d–f). Scanning electron microscope and stereomicroscope images of the compound eye revealed the many malformed bristles that are smaller than the normal bristles as observed previously^31-33,37^. Fusion of ommatidia was also observed in some regions of the compound eye compared with control flies expressing *GFP* (Fig. 1a–c). Severer eye phenotype was observed at 28 °C (Fig. 1g–i). The number of malformed small bristles increased and fusion of ommatidia was observed in a wider area in the compound eye (Fig. 1g–i). Loss of the pigment phenotype was also evident at 28 °C (Fig. 1i). Here, we thus confirmed the eye phenotype of the flies expressing *hFUS*.

### Co-expression of *NPM*-*hMLF1* suppresses the aberrant eye morphology phenotype induced by *hFUS*

**Figure 2:**
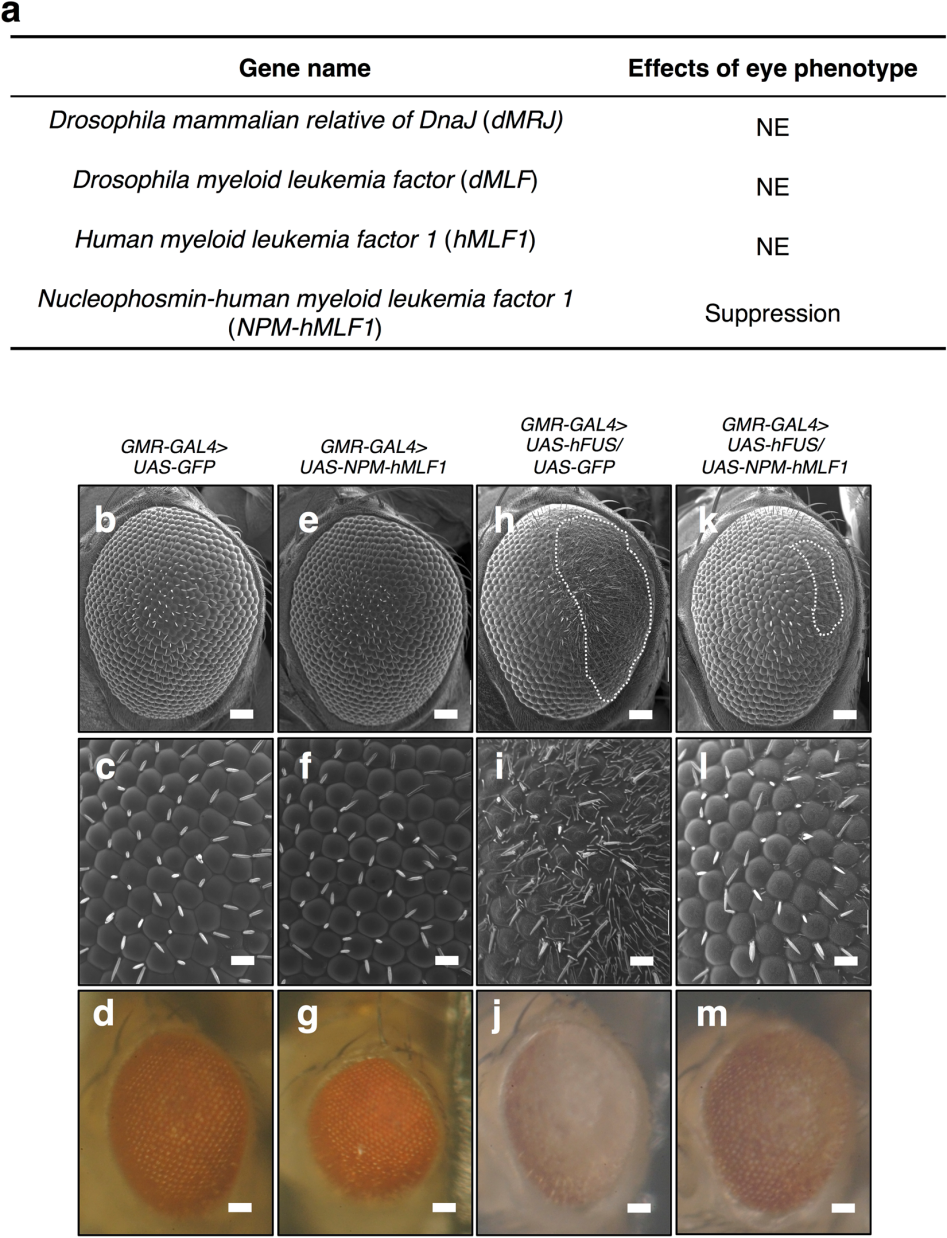
Co-expression of *NPM*-*hMLF1* suppressed aberrant eye morphology induced by *hFUS*. (**a**) Effects on aberrant eye morphology phenotype induced by *hFUS*. NE indicates no effect on eye phenotype in adults. Scanning (**b**,**c**,**e**,**f**,**h**,**i**,**k**,**l**) and stereoscopic (**d**,**g**,**j**,**m**) micrographs of adult compound eyes. (**b**–**d**) *GMR-GAL4* > *UAS-GFP* (*GMR*-*GAL4*/*Y*; *UAS*-*GFP*/+). (**e**–**g**) *GMR-GAL4* > *UAS-NPM-hMLF1* (*GMR*-*GAL4*/*Y*; *UAS*-*NPM*-*hMLF1*/+). (**h**–**j**) *GMR-GAL4* > *UAS-hFUS/UAS-GFP* (*GMR*-*GAL4*/*Y*; *UAS*-*hFUS*/*UAS*-*GFP*). (**k**–**m**) *GMR-GAL4* > *UAS-hFUS/UAS-NPM-hMLF1* (*GMR*-*GAL4*/*Y*; *UAS*-*hFUS*/*UAS*-*NPM*-*hMLF1*). (**c**,**f**,**I**,**l**) Enlarged images of (**b**,**e**,**h**,**k**) respectively. The eye phenotype of at least five newly eclosed adult male flies of each line was examined and the experiments were done in triplicate. No significant variation in eye phenotype was observed among the five individuals. Anterior is to the left and dorsal to the top. The flies were developed at 28 °C. The bars indicate 50 μm (**b**,**d**,**e**,**g**,**h**,**j**,**k**,**m**) and 14.2 μm (**c**,**f**,**I**,**l**), respectively. White dotted lines define the area of aberrant eye morphology.

To identify genes that modify the aberrant eye morphology phenotype induced by expression of *hFUS*, we crossed the *hFUS*-overexpressing flies with flies overexpressing the following genes: *Drosophila mammalian relative DnaJ* (*dMRJ*), *Drosophila myeloid leukemia factor* (*dMLF*) and *hMLF1*. Overexpression of these genes is reported to suppress the eye degeneration phenotype of polyQ disease model flies^40,41^. However, co-expression of these three genes exhibited no apparent suppression of the aberrant eye morphology phenotype induced by *hFUS* (Fig. 2a). It is reported that dMLF mainly localizes in nucleus, although its localization changes depending on the tissue, the time of development, the cell cycle and its isoforms^46^. In contrast, it is shown that hMLF1 mainly localizes in cytoplasm in mammalian cultured cells, but it also partly localizes in nucleus^42,43,47,48^. Subcellular localization of dMRJ was not fully examined, but overexpressed dMRJ was co-localized with cytoplasmic polyQ aggregates in polyQ disease model flies^40^. We considered that the difference in subcellular localization may explain the apparent lack of expression from these proteins on the aberrant eye morphology phenotype induced by expression of *hFUS*. We therefore examined the effect of overexpression of *NPM*-*hMLF1* as it is known that this fusion protein mainly localizes in the nucleus^43^. No aberrant eye morphology induced by *NPM*-*hMLF1* was observed compared with control flies expressing *GFP* (Fig. 2b–g). Strong suppression of the aberrant eye morphology phenotype induced by *hFUS* was observed by co-expression of *NPM*-*hMLF1* (Fig. 2h–m). In the following studies, we therefore focused on the detailed mechanism for this suppression.

### NPM-hMLF1 appears to localize mainly in nucleoplasm of eye imaginal disc cells

**Figure 3:**
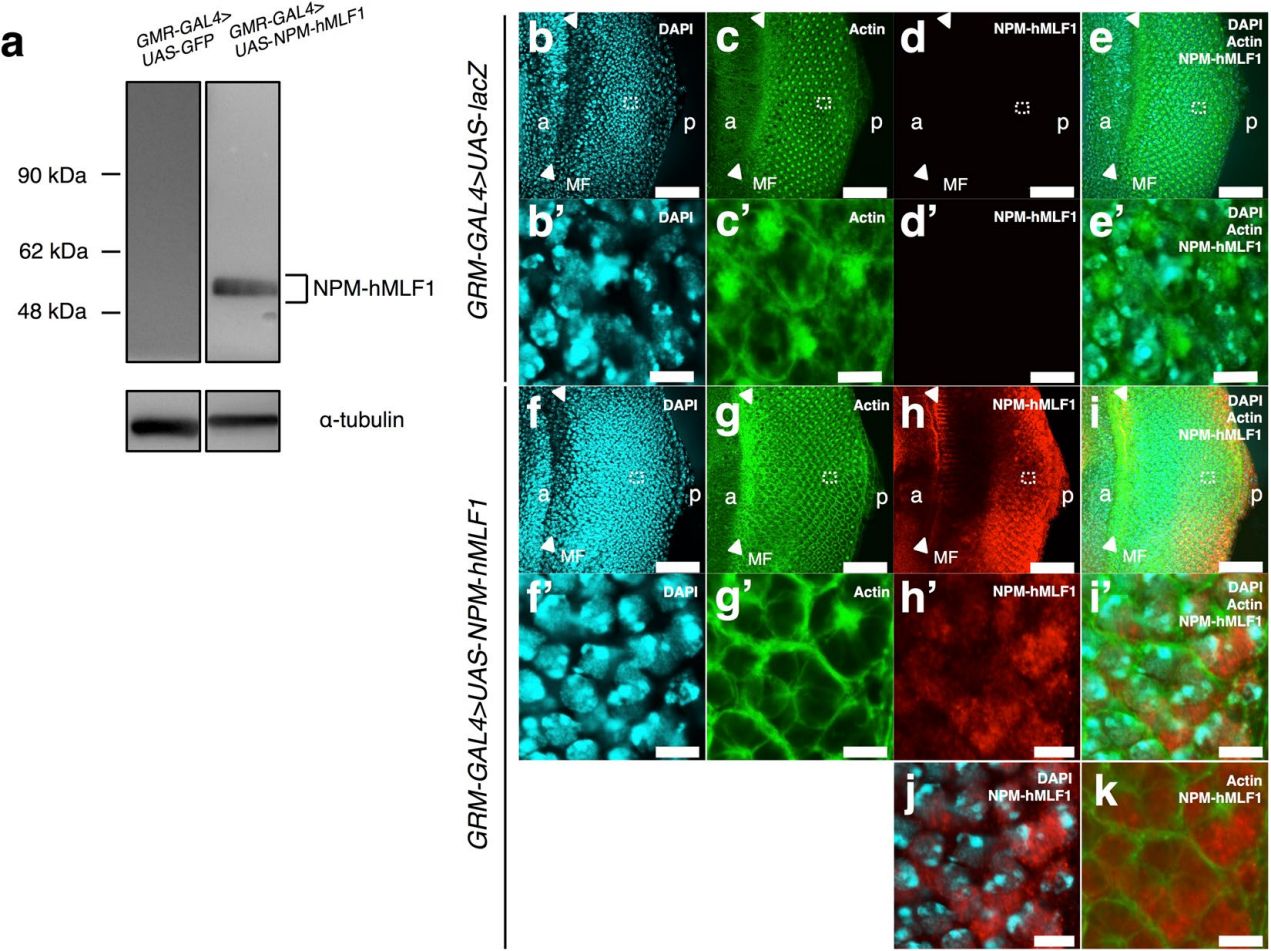
Specificity of the anti-hMLF1 IgG and immunocytochemical localization of NPM-hMLF1 in eye imaginal disc cells. (**a**) Western immunoblotting analysis. Proteins were extracted from adult heads carrying *GMR-GAL4* > *UAS-GFP* (*GMR*-*GAL4*/*Y*; *UAS*-*GFP*/+) and *GMR-GAL4* > *UAS-NPM-hMLF1* (*GMR*-*GAL4*/*Y*; *UAS*-*NPM*-*hMLF1*/+). The blots were probed with the anti-hMLF1 IgG. The band with an apparent molecular mass of 56,000 corresponds to NPM-hMLF1 protein. α-tubulin was used as a loading control. The flies were developed at 28 °C. Three independent experiments were carried out. (**b**–**k** and **b’**–**i’**) Immunostaining of eye imaginal discs with the anti-hMLF1 IgG. (**b**–**e** and **b’**–**e’**) *GMR-GAL4* > *UAS-lacZ* (*GMR*-*GAL4*/*Y*; *UAS*-*lacZ*/+). (**f**–**k** and **f’**–**i’**) *GMR-GAL4* > *UAS-NPM-hMLF1* (*GMR*-*GAL4*/*Y*; *UAS*-*NPM*-*hMLF1*/+). Eye imaginal discs were stained with DAPI (**b**,**f**,**b’** and **f’**), phalloidin (**c**,**g**,**c’** and **g’**) and anti-hMLF1 IgG (**d**,**h**,**d’** and **h’**). Merged confocal images are shown in (**e**,**i**,**j**,**k**,**e’** and **i’**). Higher magnification images of the indicated regions in the panels (**b**–**i**) are shown in (**b’**–**i’**), respectively. Arrowheads indicate MF. a indicates anterior and p indicates posterior. The flies were developed at 28 °C. Bars indicate 50 μm (**b**-**i**) and 5 μm (**j**,**k** and **b’**–**i’**).

To confirm the expression of NPM-hMLF1 fusion protein in flies carrying the *NPM*-*hMLF1* fusion gene, we performed Western immunoblotting analysis with extracts of adult heads carrying *GMR*-*GAL4*/*Y*; *UAS*-*NPM*-*hMLF1*/+. A single major band corresponding to 53 kDa was detected on the immunoblot using the anti-hMLF1 IgG, which recognized the C-terminal region of hMLF1 protein, but not with the extracts of the control flies expressing *GFP* (Fig. 3a). The reported molecular mass of NPM-hMLF1 is 56 kDa, which is generated by fusion of NPM (38 kDa) with hMLF1 (31 kDa)^43^. Thus, we confirmed the expression of NPM-hMLF1. The data also confirmed the specificity of anti-hMLF1 IgG.

Then, to examine subcellular localization of the NPM-hMLF1 fusion protein, we conducted immunostaining of eye imaginal discs with the anti-hMLF1 IgG. Strong NPM-hMLF1 signals were detected in the posterior region to MF in the eye imaginal discs (Fig. 3h). No such signal was detected in eye imaginal discs of the control flies expressing *lacZ* (Fig. 3d). Merged images showed that NPM-hMLF1 signals mainly co-localized with 4′ 6-diamidono-2-phenylindole (DAPI), especially in the DAPI faint region (Fig. 3j). The data suggest that NPM-hMLF1 mainly localizes in nucleoplasm of eye imaginal disc cells. However, since in some cells NPM-hMLF1 signals partially co-localized with cytoplasmic actin signals (Fig. 3k), we cannot exclude the possibility that some fractions of NPM-hMLF1 proteins also localize in cytoplasm.

### hFUS appears to co-localize with NPM-hMLF1 mainly in nucleoplasm of eye imaginal disc cells

**Figure 4:**
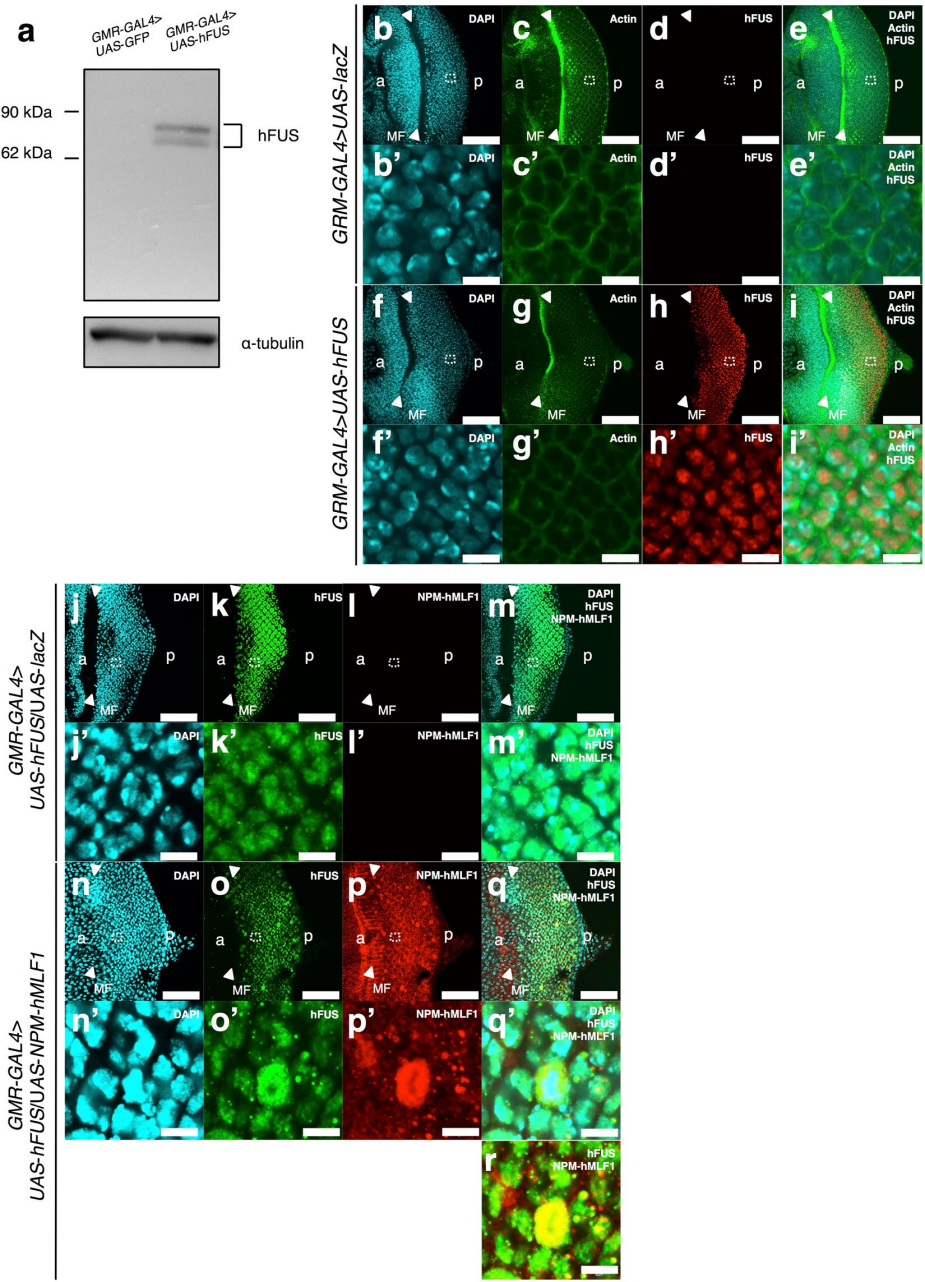
Specificity of the anti-FUS IgG and co-localization of hFUS with NPM-hMLF1 in eye imaginal disc cells. (**a**) Western immunoblotting analysis. Proteins were extracted from adult heads carrying *GMR-GAL4* > *UAS-GFP* (*GMR*-*GAL4*/*Y*; *UAS*-*GFP*/+) and *GMR-GAL4* > *UAS-hFUS* (*GMR*-*GAL4*/*Y*; *UAS*-*hFUS*/+). The blots were probed with anti-hFUS IgG. The bands with an apparent molecular mass of 83 kDa and 72 kDa correspond to hFUS protein. α-tubulin was used as a loading control. The flies were developed at 28 °C. Three independent experiments were carried out. (**b**–**i** and **b’**–**i’**) Immunostaining of eye imaginal discs with the anti-hFUS IgG. (**b**–**e** and **b’**–**e’**) G*MR-GAL4* > *UAS-lacZ* (*GMR*-*GAL4*/*Y*; *UAS*-*lacZ*/+). (**f**–**i** and **f’**–**i’**) *GMR-GAL4* > *UAS-hFUS* (*GMR*-*GAL4*/*Y*; *UAS*-*hFUS*/ + ). Eye imaginal discs were stained with DAPI (**b**,**f**,**b’** and **f’**), phalloidin (**c**,**g**,**c’** and **g’**) and anti-hFUS IgG (**d**,**h**,**d’** and **h’**). Merged confocal images are shown in (**e**,**i**,**e’** and **i’**). Higher magnification images of the indicated regions in the panels (**b**–**i**) are shown in (**b’**–**i’**) respectively. Arrowheads indicate MF. a indicates anterior and p indicates posterior. The flies were developed at 28 °C. Bars indicate 50 μm (**b**-**i**) and 5 μm (**b’**–**i’**). (**j**–**r** and **j’**–**q’**) Immunostaining of eye imaginal discs with the anti-hFUS IgG and the anti-hMLF1 IgG. (**j**–**m** and **j’**–**m’**) *GMR-GAL4* > *UAS-hFUS/UAS-lacZ* (*GMR*-*GAL4*/*Y*; *UAS*-*hFUS*/*UAS*-*lacZ*+) (**n**–**r** and **n’**–**q’**) G*MR-GAL4* > *UAS-hFUS/UAS-NPM-hMLF1* (*GMR*-*GAL4*/*Y*; *UAS*-*hFUS*/*UAS*-*NPM*-*hMLF1*). Eye imaginal discs were stained with DAPI (**j**,**n**,**j’** and **n’**), anti-hFUS IgG (**k**,**o**,**k’** and **o’**) and anti-hMLF1 IgG (**l**,**p**,**l’** and **p’**). Merged confocal images are shown in **m**,**q**,**r**,**m’** and **q’**. Higher magnification images of the indicated regions in the panels (**j**–**q**) are shown in (**j’**–**q’**) respectively. Arrowheads indicate MF. a indicates anterior and p indicates posterior. The flies were developed at 28 °C. Bars indicate 50 μm (**j**–**q**) and 5 μm (**r** and **j’**–**q’**).

To confirm specificity of anti-hFUS IgG, we performed Western immunoblotting analysis with extracts of adult heads carrying *GMR*-*GAL4*/*Y*; *UAS*-*hFUS*/+. Two major bands corresponding to 83 kDa and 72 kDa were detected on the immunoblot using the anti-hFUS IgG, but not with the extracts of the control flies expressing *GFP* (Fig. 4a). The molecular mass of these bands is within a similar range predicted from the amino acid sequences of hFUS (75 kDa). The two hFUS-immunopositive bands with similar mass were reported previously^35^. Here, we confirmed the specificity of anti-hFUS IgG.

To examine subcellular localization of hFUS protein in eye imaginal discs, we performed immunostaining with anti-hFUS IgG. Strong hFUS signals were detected in the posterior region to MF in the eye imaginal discs (Fig. 4h). No such signal was detected in eye imaginal discs of the control flies expressing *lacZ* (Fig. 4d). Merged images showed that hFUS signals co-localized with DAPI, especially in the DAPI faint region (Fig. 4I’). The data indicate that hFUS likely localizes in nucleoplasm.

In addition, we also performed double immunostaining of eye imaginal discs from flies co-expressing *hFUS* and *NPM*-*hMLF1* with the anti-hFUS IgG and anti-hMLF1 IgG. Merged images indicated that hFUS signals co-localize with NPM-hMLF1, mainly in the DAPI faint region (Fig. 4r,q’), suggesting that hFUS co-localizes with NPM-hMLF1 mainly in the nucleoplasm of eye imaginal disc cells. However, since some merged signals of hFUS and NPM-hMLF1 also localized outside of DAPI -stained region (Fig. 4q’), we cannot exclude the possibility that some fractions of hFUS and NPM-hMLF1 proteins also localize in cytoplasm.

### The driving of *hFUS* expression at 28 °C down-regulates levels of hFUS and endogenous caz proteins

**Figure 5:**
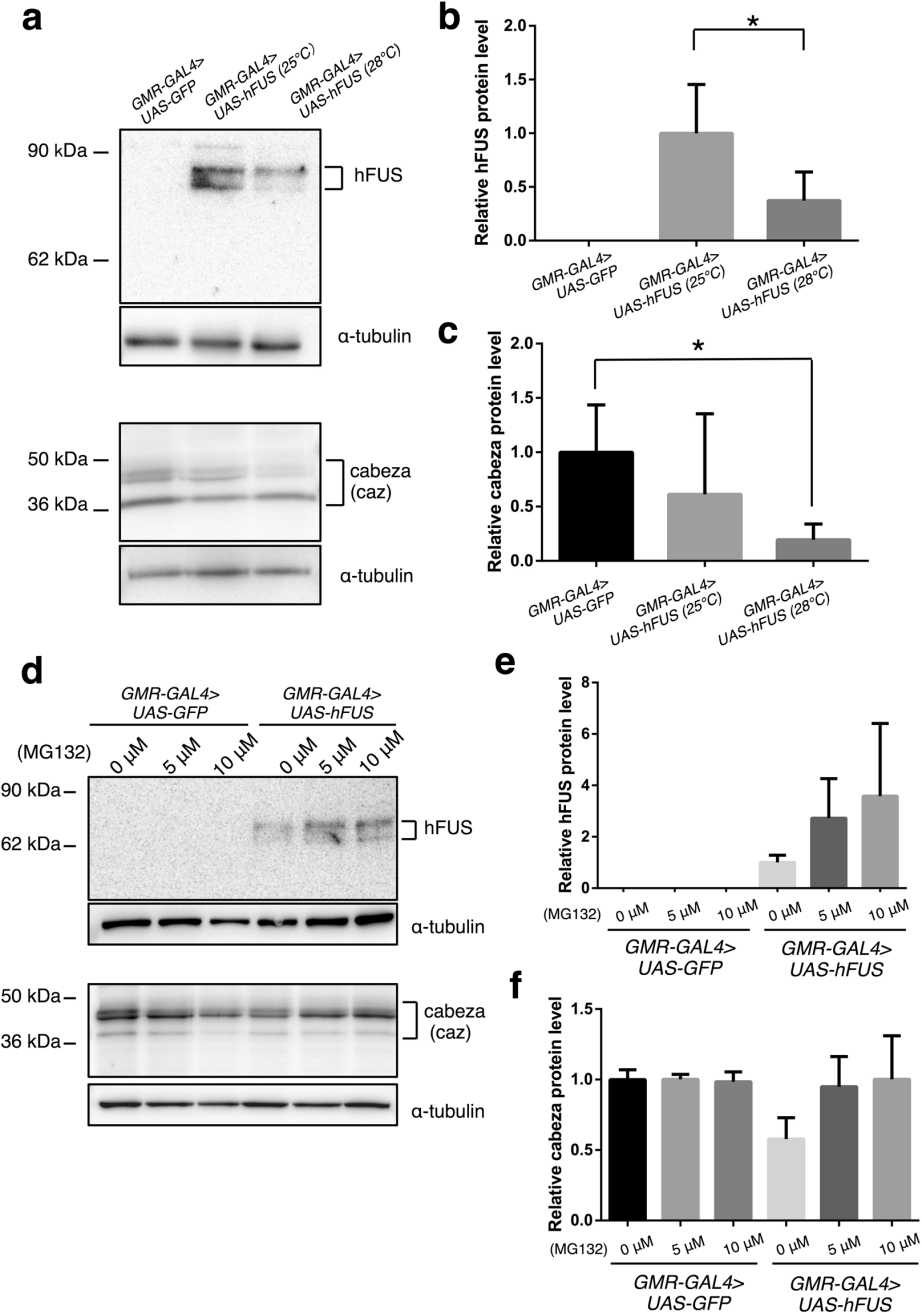
The high level of *hFUS* expression down-regulated levels of hFUS and endogenous caz by proteasome dependent degradation in the cytoplasm. (**a**) Western immunoblotting analysis. Proteins were extracted from adult heads carrying *GMR-GAL4* > *UAS-GFP* (*GMR*-*GAL4*/*Y*; *UAS*-*GFP*/+) and *GMR-GAL4* > *UAS-hFUS* (*GMR*-*GAL4*/*Y*; *UAS*-*hFUS*/+). The blots were probed with anti-hFUS IgG or anti-caz IgG. The bands with an apparent molecular mass of 83 kDa and 72 kDa correspond to hFUS protein (upper panel). The bands with molecular mass of 45 kDa and 39 kDa correspond to two caz isoforms as reported previously (lower panel)^29,30^. The flies were developed at either 25 °C or 28 °C. α-tubulin was used as a loading control. (**b**) Quantification of relative hFUS protein level in three independent experiments (n = 3). hFUS protein levels were normalized with α-tubulin and shown relative to the one at *GMR-GAL4* > *UAS-hFUS* (25 °C). The Student *t*-test was used to compare measures between two groups. *Indicates *p* < 0.05. Error bars indicate SEM. (**c**) Quantification of relative caz protein levels that were normalized with α-tubulin and shown relative to the one at *GMR-GAL4* > *UAS-GFP*. Three independent experiments were carried out (n = 3). One-way ANOVA, followed by Tukey test, was used to compare measures among three groups. *Indicates *p* < 0.05. Error bars indicate SEM. (**d**) Western immunoblotting analysis. Proteins were extracted from adult heads carrying *GMR-GAL4* > *UAS-GFP* (*GMR*-*GAL4*/*Y*; *UAS*-*GFP*/+) and *GMR-GAL4* > *UAS-hFUS* (*GMR*-*GAL4*/*Y*; *UAS*-*hFUS*/+). The blots were probed with anti-hFUS IgG or anti-caz IgG. The bands with an apparent molecular mass of 83 kDa and 72 kDa correspond to hFUS protein (upper panel). The bands with molecular mass of 45 kDa and 39 kDa correspond to two caz isoforms (lower panel)^29,30^. The flies were fed with fly food containing 0 μM, 5 μM or 10 μM of MG132 at 28 °C. α-tubulin was used as a loading control. (**e**) Quantification of relative hFUS protein level in two independent experiments (n = 2). hFUS protein levels were normalized with α-tubulin and shown relative to the one at *GMR-GAL4* > *UAS-hFUS* (0 μM). Error bars indicate SEM. (**f**) Quantification of relative caz protein level in two independent experiments (n = 2). caz protein levels were normalized with α-tubulin and shown relative to the one at *GMR-GAL4* > *UAS-GFP* (0 μM). Error bars indicate SEM.

Previous studies reported that ectopic expression of *hFUS* reduced endogenous caz^35,37^. Therefore, we re-examined the level of caz together with hFUS by Western immunoblotting analysis with extracts of adult heads carrying *GMR*-*GAL4*/*Y*; *UAS*-*hFUS*/+ developed at either 25 °C or 28 °C. At 25 °C, expression of hFUS was confirmed as expected and the level of endogenous caz was decreased to 61 percent compared to the control flies expressing *GFP* (Fig. 5a–c). Normally higher expression of hFUS is expected at 28 °C due to higher activity of the transcription factor GAL4 at 28 °C than at 25 °C^49^. However, surprisingly, not only endogenous caz level, but also hFUS level decreased to 11 percent and 37 percent at 28 °C compared with those at 25 °C, respectively (Fig. 5a–c). The data suggest a possible negative feedback mechanism that may be mediated by protein degradation at 28 °C.

### Down-regulation of both hFUS and caz are likely mediated by proteasome dependent degradation

To examine the possibility that the degradation of both hFUS and caz proteins are mediated by proteasome dependent degradation, we added proteasome inhibitor (MG132) in fly food and flies were reared at 28 °C. Interestingly, dosage-dependent feeding of MG132 from 5 μM to 10 μM inhibited both hFUS and caz protein degradation (Fig. 5d–f). The data suggest that the down-regulation of both hFUS and caz are mediated by proteasome-dependent degradation.

### Co-expression of *NPM*-*hMLF1* recovered the hFUS protein level but not caz protein level

**Figure 6:**
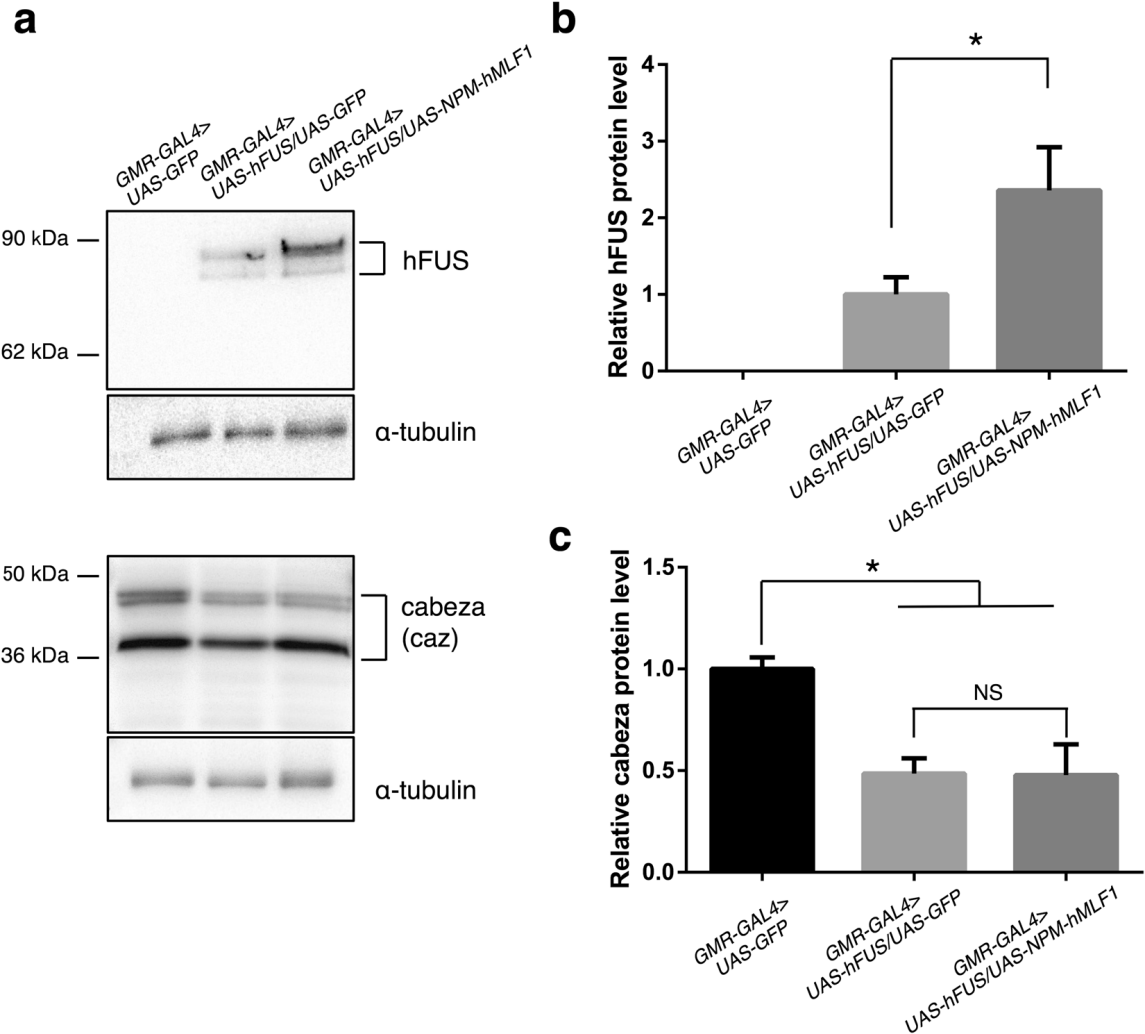
Co-expression of *NPM*-*hMLF1* supressed hFUS protein degradation. (**a**) Western immunoblotting analysis. Proteins were extracted from adult heads carrying *GMR-GAL4* > *UAS-GFP* (*GMR*-*GAL4*/*Y*; *UAS*-*GFP*/+), *GMR-GAL4* > *UAS-hFUS/UAS-GFP* (*GMR*-*GAL4*/*Y*; *UAS*-*hFUS*/*UAS*-*GFP*) and *GMR-GAL4* > *UAS-hFUS/UAS-NPM-hMLF1* (*GMR*-*GAL4*/*Y*; *UAS*-*hFUS*/*UAS*-*NPM*-*hMLF1*). The blots were probed with anti-hFUS IgG or anti-caz IgG. The bands with an apparent molecular mass of 83 kDa and 72 kDa correspond to hFUS (upper panel). The bands with molecular mass of 45 kDa and 39 kDa correspond to caz two isoforms (lower panel)^29,30^. The flies were developed at 28 °C. α-tubulin was used as a loading control. (**b**) Quantification of relative hFUS protein level in three independent experiments (n = 3). hFUS protein levels were normalized with α-tubulin and shown relative to the one at *GMR-GAL4* > *UAS-hFUS/UAS-GFP*. Student *t*-test was used to compare measures between two groups. *Indicates *p* < 0.05. Error bars indicate SEM. (**c**) Quantification of relative caz protein level that were normalized with α-tubulin and shown relative to the one at *GMR-GAL4* > *UAS-GFP*. Three independent experiments were carried out (n = 3). One-way ANOVA, followed by Tukey test, was used to compare measures among three groups. *Indicates *p* < 0.05. Error bars indicate SEM.

To examine the effect of co-expression of *NPM*-*hMLF1* on levels of hFUS and caz proteins at 28 °C, we performed Western immunoblotting analysis with extracts of adult heads carrying *GMR*-*GAL4*/*Y*; *UAS*-*hFUS*/*UAS*-*GFP* and *GMR*-*GAL4*/*Y*; *UAS*-*hFUS*/*UAS*-*NPM*-*hMLF1*. Expression of hFUS decreased caz protein level by 51 percent (Fig. 6a,c), which corelates with the eye phenotype (Fig. 2h–j). Co-expression of *NPM*-*hMLF1* increased hFUS protein level approximately two fold, but the decreased caz protein level was not recovered (Fig. 6a–c). The eye phenotype was suppressed under these conditions (Fig. 2k–m). These results indicate that co-expression of *NPM*-*hMLF1* could suppress hFUS degradation but not caz degradation. The data implicate that decreased caz level is critical to induce the eye phenotype (Fig. 2h–j), and that the caz function may be partially complemented by an increased level of hFUS (Fig. 2k–m)^30^.

### Co-expression of *NPM*-*hMLF1* slightly increased the solubility of hFUS protein

**Figure 7:**
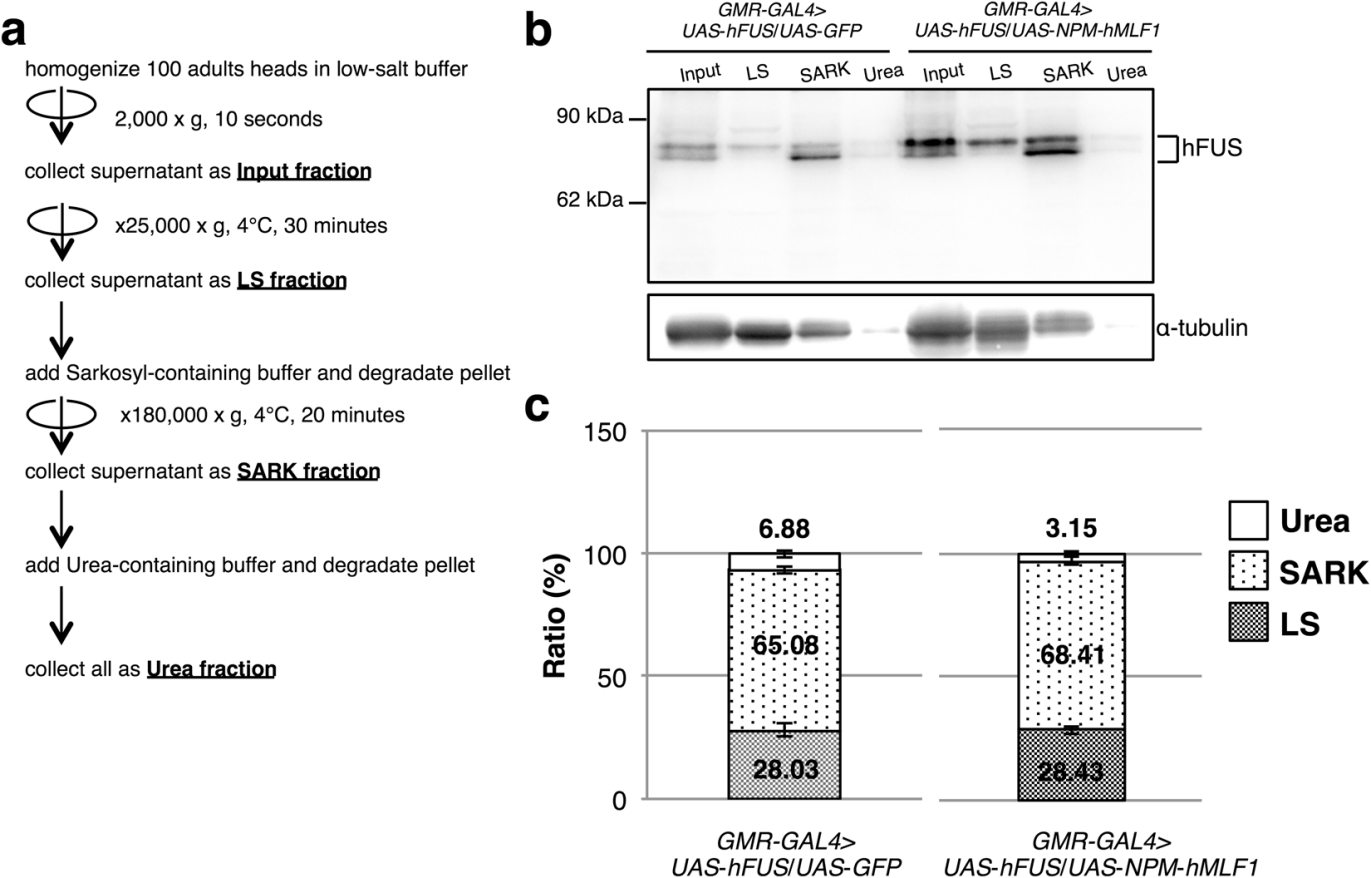
Co-expression of *NPM*-*hMLF1* slightly increased the solubility of hFUS protein. (**a**) Schematic drawings of protocol for proteins that were fractionated into Input fraction, LS fraction, SARK fraction and Urea fraction. (**b**) Western immunoblotting analysis of proteins recovered in each fraction with hFUS IgG. Proteins were extracted from adult heads carrying *GMR-GAL4* > *UAS-hFUS/UAS-GFP* (*GMR*-*GAL4*/*Y*; *UAS*-*hFUS*/*UAS*-*GFP*) and *GMR-GAL4* > *UAS-hFUS/UAS-NPM-hMLF1* (*GMR*-*GAL4*/*Y*; *UAS*-*hFUS*/*UAS*-*NPM*-*hMLF1*). After fractionating by the above protocol, samples were analyzed by Western blotting. The blots were probed with anti-hFUS IgG. The bands with an apparent molecular mass of 83 kDa and 72 kDa correspond to hFUS protein. The flies were developed at 28 °C. The α-tubulin was used as a loading control. (**c**) Quantification of hFUS protein level in each of the LS, SARK and Urea fractions from the two independent experiments (n = 2). The amounts of hFUS protein in the LS fraction, SARK fraction and Urea fraction were normalized to Input fraction and shown as a relative ratio (%). Error bars indicate SEM.

In transgenic flies carrying mutant or wild-type *hFUS*, there is no obvious evidence showing apparent FUS-immunopositive inclusions^31–37^. To further confirm these previous observations obtained by cytological and biochemical analyses, we examined a possible formation of FUS-immunopositive aggregates. We performed solubility tests on hFUS protein with extracts of adult heads carrying *GMR*-*GAL4*/*Y*; *UAS*-*hFUS*/*UAS*-*GFP* developed at 28 °C as summarized in Fig. 7a. This solubility test has been used to evaluate protein aggregates containing human TDP-43 (hTDP-43) protein in *Drosophila* cells^50^. Nearly 28 (mean; 28.03 ± 2.78) percent of the Input hFUS proteins in extracts of adult heads were recovered in low salt soluble fraction (LS) (Fig. 7b,c). Nearly 65 (mean; 65.08 ± 1.21) percent of the Input hFUS proteins were recovered in low salt insoluble and 1 percent Sarkosyl soluble fraction (SARK) (Fig. 7b,c). Nearly 7 (mean; 6.88 ± 1.58) percent of the Input hFUS proteins were recovered in 1 percent Sarkosyl insoluble and 7 M Urea soluble fraction (Urea) (Fig. 7b,c). No hFUS proteins were recovered in 7 M Urea insoluble fraction. Here, we thus confirmed the biochemical property of hFUS protein in *Drosophila* cells.

To examine the effects of co-expression of *NPM*-*hMLF1* on the solubility of hFUS protein, we carried out solubility tests with extracts of adult heads of flies co-expressing *hFUS* and *NPM*-*hMLF1* developed at 28 °C. Nearly 28 (mean; 28.43 ± 1.54) percent of the Input hFUS proteins were recovered in LS fraction (Fig. 7b,c). Nearly 68 (mean; 68.41 ± 1.97) percent of the Input hFUS proteins were recovered in SARK fraction (Fig. 7b,c). Nearly 3 (mean; 3.15 ± 0.42) percent of the Input hFUS proteins were recovered in Urea fraction (Fig. 7b,c). Here, we thus found that co-expression of *NPM*-*hMLF1* slightly increased the solubility of hFUS protein.

### Co-expression of *NPM-hMLF1* partially rescued pharate adult lethal phenotype induced by motor neuron-specific expression of *hFUS*

**Figure 8:**
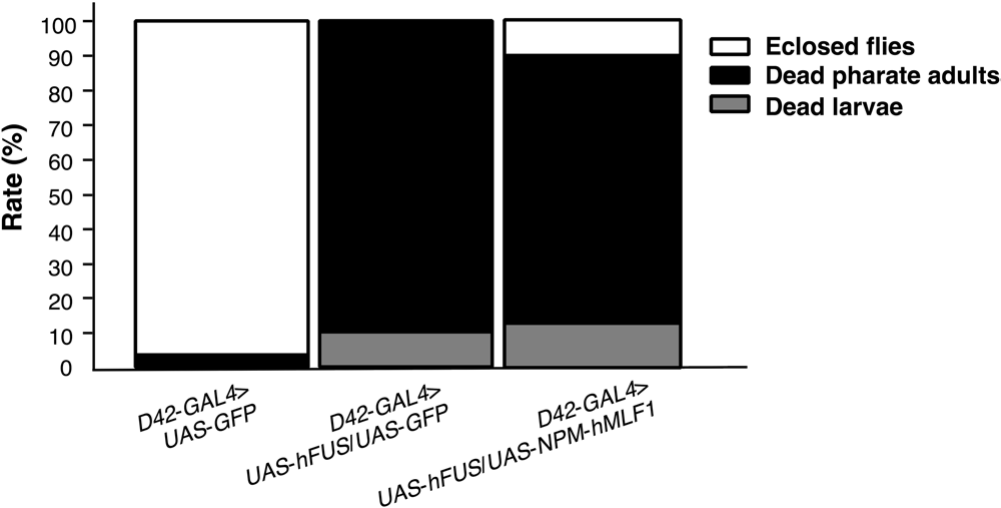
Co-expression of *NPM*-*hMLF1* partially rescued the pharate adult lethal phenotype induced by motor neuron-specific expression of *hFUS*. Assessment of eclosion rate, pupal lethality and larval lethality. First larvae carrying *w*; *UAS*-*GFP*/+; *D42*-*GAL4*/+, *w*; *UAS*-*hFUS*/*UAS*-*GFP*; *D42*-*GAL4*/+ and *w*; *UAS*-*hFUS*/*UAS*-*NPM*-*hMLF1*; *D42*-*GAL4*/+ were collected. Ten larvae were reared at 25 °C in a single plastic vial containing fly food. The number of eclosed adult flies, the number of dead pupae that were unable to eclose, and the number of dead larvae that were unable to reach the pupal stage were counted. Eclosed adult flies (48/50), dead pharate adults (2/50) and dead larvae (0/50) were observed in flies carrying *w*; *UAS*-*GFP*/+; *D42*-*GAL4*/+ (n = 50). Eclosed adult flies (0/42), dead pharate adults (38/42) and dead larvae (4/42) were observed in flies carrying *w*; *UAS*-*hFUS*/*UAS*-*GFP*; *D42*-*GAL4*/+ (n = 42). Eclosed adult flies (4/41), dead pharate adults (32/41) and dead larvae (5/41) were observed in flies carrying *w*; *UAS*-*hFUS*/*UAS*-*NPM*-*hMLF1*; *D42*-*GAL4*/+ (n = 41).

To examine the effect of co-expression of *NPM*-*hMLF1* on pharate adult lethal phenotype induced by *hFUS* in motor neurons, we examined the viability of flies carrying *w*; *UAS*-*GFP*/+; *D42*-*GAL4*/+, *w*; *UAS*-*hFUS*/*UAS*-*GFP*; *D42*-*GAL4*/+ and *w*; *UAS*-*hFUS*/*UAS*-*NPM*-*hMLF1*; *D42*-*GAL4*/+ and then calculated the rate of eclosed adult flies, dead pharate adults and dead larvae. Co-expression of *NPM*-*hMLF1* partially suppressed pharate adult lethal phenotype induced by *hFUS* (Fig. 8). No eclosion was observed with flies carrying *UAS*-*hFUS*/*UAS*-*GFP*; *D42*-*GAL4*/+. Nearly 100 percent eclosion was observed with control flies expressing *GFP* under control of *D42 GAL4*. The eclosion rate increased to approximately 10 percent by co-expression of *NPM*-*hMLF1*. The data indicate that co-expression of *NPM*-*hMLF1* partially rescued the defect induced by *hFUS* in motor neurons.

## Discussion

In the present study, we observed a strong eye phenotype at 28 °C. Unexpectedly, the hFUS level decreased at 28 °C compared to 25 °C and this hFUS reduction was inhibited by feeding a proteasome inhibitor to flies. The data suggest that the strong eye phenotype at 28 °C is likely due to proteasome-dependent degradation of hFUS protein. Interestingly, co-expression of *NPM*-*hMLF1* suppressed the eye phenotype with the increase in hFUS protein level, but not caz. In addition, we also revealed that co-expression of *NPM*-*hMLF1* partially rescued pharate adult lethal phenotype induced by motor neuron-specific expression of *hFUS*, implying that NPM-hMLF1 may modify FUS-related FTLD pathology.

Negative feedback mechanisms associated with hFUS were reported^51,52^. For instance, it was reported that hFUS binds to the exon 7 region of its own gene to inhibit splicing of *hFUS* mRNA that results in reduction of hFUS expression^51^. The other study revealed a different negative feedback mechanism that is mediated by repressive miRNAs, such as miR-141 and miR-200a that hybridize to 3′ UTR of *hFUS* mRNA^52^. In addition to these, here we suggest a novel proteasome-dependent negative feedback mechanism of hFUS. It was reported that hFUS can be transported from cytoplasm to the nucleus that is mediated by transportin1^17^. Previous studies indicate that tight binding of transportin1 with hFUS in the nucleus leads to re-export of this complex to cytoplasm in FTLD pathology^53,54^. By this mechanism, this complex accumulates in cytoplasm, resulting in the depletion of hFUS in the nucleus^53,54^. Based on these reports, we can propose a negative feedback model on hFUS protein stability indicating that overexpression of hFUS may activate both this transportation system and proteasome-dependent depletion of hFUS. The accumulated hFUS in cytoplasm may also be degraded by proteasome and consequently hFUS in the nucleus can be depleted. Further analysis is necessary to clarify a detailed mechanism.

In the present study, it was shown that the co-expression of three genes (*dMRJ*, *dMLF* and *hMLF1*) exerts no effect on the eye phenotype induced by *hFUS*. Interestingly, both hFUS and NPM-hMLF1 were found to be co-localized in nucleoplasm of *Drosophila* eye imaginal disc cells in the flies co-expressing *hFUS* and *NPM*-*hMLF1*. Therefore, co-localization of NPM-hMLF1 with hFUS in nucleoplasm is likely critical for the suppression of the aberrant eye morphology phenotype induced by *hFUS*. It was reported that dMLF directly binds to RUNX transcription factor Lozenge and regulates the stability of Lozenge in a proteasome-dependent manner^55–57^. It should also be noted that NPM co-immunoprecipitates with hFUS in human hepatocellular carcinoma, HepG2 cells^58^. Considering these findings, NPM-hMLF1 possibly binds to hFUS to protect from degradation.

Pathological features in FTLD/ALS are represented by formation of neuronal and glial inclusions. TDP-43 is another DNA/RNA binding protein that is contained in the inclusion observed in FTLD patients similar to FUS^59^. Biochemical studies revealed that inclusions containing hTDP-43 in FTLD patients tissue are recovered in Urea soluble fractions^60^. Moreover, previous studies showed the evidence of Urea soluble hTDP-43 protein in transgenic flies expressing wild-type *hTDP-43*^50^. In contrast, inclusions containing hFUS are recovered in sodium dodecyl sulfate (SDS) fractions in tissues of human FTLD patients^10^. Here, we observed a higher level of Sarkosyl soluble hFUS protein than that of Urea soluble hFUS protein in transgenic flies expressing *hFUS*, suggesting that hFUS is less insoluble than hTDP43 in *Drosophila* cells as observed with human FTLD patients. In addition, it was also reported that increasing the solubility of hTDP-43 protein by co-expression of *futsch* closely correlates with extension of life span and improvement of locomotive defects in flies expressing *hTDP-43*^50^. Similarly to this report, we found that co-expression of *NPM*-*hMLF1* slightly increased the solubility of hFUS protein. Furthermore, eye phenotype and pharate adult lethal phenotype induced by *hFUS* were suppressed by co-expression of *NPM*-*hMLF1*. These data indicate that refolding of hFUS protein aggregates by *NPM*-*hMLF1* can be applied to novel therapy in human FTLD pathology.

## Methods

### Fly stocks

Fly stocks were maintained at 25 °C on standard food containing 0.7% agar, 5% glucose and 7% dry yeast. Canton S was the used as the wild type. *w*^1118^; *P [UAS-GFP. nls]*^2^, *w*; *P[GawB]* ^*D4*2^, *w*; *P[UAS-dmrj.Flag]*^2^, *w*; *P[UAS-hMLF1.FLAG]*^*2*^ and *w*^1118^; *P[UAS-dMLF.FLAG]*^3^ were obtained from Bloomington *Drosophila* Stock Center. *w*; *P[UAS-lacZ]*^2^ were obtained from the Kyoto Stock Center. The transgenic fly lines carrying *GMR*-*GAL4* were as described earlier^61,62^.

### Establishment of transgenic flies

This method was adopted from a protocol described previously^63–65^. pUAST-*NPM*-*hMLF1* were constructed by inserting the NPM-MLF1 cDNA into the pUAST and then injected into embryos to obtain a stable transformant line carrying *UAS*-*NPM*-*hMLF1*^42,43^. P-element-mediated germline transformation was performed. F1 transformants were selected based on white eye color rescue. To generate pUAST-*hFUS* vectors, we used the Gateway^®^ Vector Conversion System (Thermo Fisher Scientific). The human *FUS* cDNA was subcloned into the pENTR™/D-TOPO^®^ vector (Thermo Fisher Scientific). To generate the Gateway destination vector pUAST-DEST, we inserted the Gateway cassette A sequence (Thermo Fisher Scientific) into the pUAST vector. The pUAST-*hFUS* vector was generated using Gateway recombination reactions (Thermo Fisher Scientific). To establish transgenic flies harboring *UAS-hFUS*, the pUAST-*hFUS* vector was injected into fly embryos using standard methods, by BestGene Inc.

### Western immunoblotting analysis

Protein extracts from one hundred adult heads were prepared. Briefly, the adult heads were homogenized in a sample buffer containing 50 mM Tris-HCl (pH 6.8), 2% SDS, 10% glycerol, 0.1% bromophenol blue and 1.2% β-mercaptoethanol. The homogenates were incubated at 95 °C for 3 minutes, and then centrifuged. The supernatants were separated by electrophoresis on SDS-polyacrylamide gels containing 8% or 12% acrylamide and then the polypeptides were transferred to polyvinylidene difluoride membranes (Bio-Rad). The blotted membranes were subsequently blocked with TBS/0.3% Tween 20 containing 0.1% skim milk for 1 hour at 25 °C, and incubated with primary antibody for 16 hours at 4 °C. The following primary antibodies were used: mouse anti-FUS IgG at a 1:200 dilution (Santa-Cruz), rabbit anti-caz IgG at a 1:800 dilution and rabbit anti-hMLF1 IgG at a 1:200 dilution (Santa-Cruz)^30^. After extensive washing, the membranes were incubated with HRP-conjugated anti-mouse IgG (GE healthcare) or HRP-conjugated anti-rabbit IgG (GE healthcare) at a 1:10,000 dilution for 2 hours at 25 °C. The bound antibody was detected using ECL-select Western blotting detection reagents (GE healthcare) and images were analyzed with AE-9300H Ez-Capture MG (ATTO). To ensure equal protein loading in each lane, the membranes were also probed with an anti-α-tubulin IgG after stripping the complex of primary antibody and HRP-conjugated anti-mouse IgG or HRP-conjugated anti-rabbit IgG. For the detection of α-tubulin, mouse anti-α-tubulin monoclonal IgG at a 1:5,000 dilution (Sigma-Aldrich) and HRP-conjugated anti-mouse IgG at a 1:10,000 dilution (GE healthcare) were used as the primary and secondary antibodies, respectively.

### Solubility test

This method was adopted from a protocol described previously^50^. Briefly, one hundred adult heads were homogenized in 300 μl of low-salt buffer (10 mM Tris-HCl, 5 mM EDTA, 10% sucrose, pH 7.5). Homogenates were centrifuged at 2,000 × g for 10 seconds. After discarding cellular debris and cuticle, 70 μl of the homogenate was set aside as Input fraction. The remaining 230 μl of homogenate was centrifuged at 25,000 × g for 30 minutes at 4 °C. The supernatant from the solution was set aside as LS fraction. The pellet was then extracted with Sarkosyl-containing buffer (10 mM Tris, 5 mM EDTA, 1% Sarkosyl, 10% sucrose, pH 7.5) and centrifuged at 180,000 × g for 20 minutes at 4 °C. The supernatant from the solution was set aside and designated as SARK fraction. The remaining Sarkosyl-insoluble pellet was then solubilized in Urea-containing buffer (30 mM Tris, 7 M Urea, 2 M Thiourea, 4% CHAPS, pH 8.5). The recovered fraction was named the Urea fraction. Protein concentrations were determined and 20 μg of each fraction was loaded on 8 or 12% SDS-PAGE. All buffers contained 2 × Complete Protease Inhibitor Cocktail (Roche) supplemented with 0.5 mM phenylmethylsulfonylfluoride to inhibit proteolysis and were filtered through 0.45 μm filter.

### Immunostaining

For immunohistochemistry, eye imaginal discs from larvae were dissected and fixed in 4% paraformaldehyde/PBS for 30 minutes at 25 °C. These tissue samples were washed with PBS containing 0.3% Triton X-100; fixed samples were then incubated with Alexa 488-conjugated phalloidin (1unit/200 μl) (Thermo Fisher Scientific) in PBS containing 0.3% Triton X-100 for 20 minutes at 25 °C. The samples were then blocked with PBS containing 0.15% Triton X-100 and 10% normal goat serum (blocking buffer) for 30 minutes at 25 °C and incubated with primary antibodies in PBS containing 0.15% Triton X-100 and 10% normal goat serum for 16 hours at 4 °C. The following antibodies were used; mouse anti-FUS IgG at a 1:50 dilution (Santa-Cruz) and rabbit anti-hMLF1 IgG at a 1:50 dilution (Santa-Cruz). After washing with PBS containing 0.3% Triton X-100, the samples were stained with DAPI (0.5 μg/ml) (Thermo Fisher Scientific) in PBS containing 0.1% Triton X-100. After extensive washing with PBS containing 0.3% Triton X-100, samples were incubated with secondary antibodies labeled with Alexa 488 or Alexa 594 at a 1:400 dilution (Invitrogen) for 3 hours at 25 °C. After extensive washing with PBS containing 0.3% Triton X-100, samples were mounted in the Vectashield (Vector laboratories), and inspected using a confocal laser-scanning microscope (Olympus Fluoview FV10i).

### Proteasome inhibition

This method was adopted from a protocol described previously^66^. Flies were reared at 28 °C in a single plastic vial containing 5 mL of *Drosophila* instant medium (Formula 4–24, Blue, Carolina Biological Supply Company) with or without indicated amount of MG132 (Sigma-Aldrich). Briefly, one hundred newly eclosed adult flies were collected. In parallel, individual flies were used for Western immunoblotting analysis.

### Scanning electron microscopy and stereomicroscopy

Adult flies were anesthetized, mounted and inspected under a scanning electron microscope (Keyence VE-7800) in high vacuum mode and stereomicroscope (Olympus SZX-ILLB100). In every experiment, the eye phenotypes of at least five newly eclosed adult male flies of each line were simultaneously examined by scanning electron microscope and stereomicroscope. Three independent experiments for each line were carried out and no significant variation in the compound eye phenotype was observed among individuals.

### Viability assay

This method was adopted from a protocol described previously^67,68^. Briefly, at least thirty first instar larvae were collected and transferred to fresh food. After eclosion, the rate of eclosed adult flies, dead pharate adults and dead larvae were calculated.

### Data analysis

GraphPad Prism version 6.02 was used for statistics analysis. Student *t*-test was used to statistically compare between two independent groups. One-way ANOVA followed by Tukey test was used to statistically compare measures among three groups. The significance between the variables was described based on the *p*-value: NS indicates *p* > 0.05, *Indicates *p* < 0.05. Values are described as means and error bars indicate standard error means (SEM).

### Data availability

The datasets generated during and/or analyzed during the current study are available from the corresponding author on reasonable request.

## Electronic supplementary material

Supplementary information
