# Supplementary material for: NPM-hMLF1 fusion protein suppresses defects of a Drosophila FTLD model expressing the human FUS gene
Source: Sci Rep. 2018 Jul 26;8:11291. doi: 10.1038/s41598-018-29716-9 (PMC6062494; doi:10.1038/s41598-018-29716-9)
Supplement: Supplementary file 1 — Supplementary information [file 41598_2018_29716_MOESM1_ESM.pdf]

Supplementary information

NPM-hMLF1 fusion protein suppresses defects of a *Drosophila* FTLD model expressing the human *FUS* gene

Itaru Yamamoto[1,2], Yumiko Azuma[3], Yukie Kushimura[3], Hideki Yoshida [1,2], Ikuko Mizuta[3], Toshiki Mizuno[3], Morio Ueyama[5], Yoshitaka Nagai[5], Takahiko Tokuda[3,4] and Masamitsu Yamaguchi[1,2*]

[1]Department of Applied Biology and [2]The Center for Advanced Insect Research, Kyoto Institute of Technology, Matsugasaki, Sakyo-ku, Kyoto 606-8585, Japan; [3]Department of Neurology and [4]Department of Molecular Pathobiology of Brain Diseases, Graduate School of Medical Science, Kyoto Prefectural University of Medicine, 465 Kajii-cho, Kamigyo-ku, Kyoto 602-8566, Japan; [5]Department of Neurotherapeutics, Osaka University Graduate School of Medicine, 2-2 Yamadaoka, Suita, Osaka 565-0871, Japan.

Correspondence: M. Yamaguchi, Department of Applied Biology, Kyoto Institute of Technology, Matsugasaki, Sakyo-ku, Kyoto 606-8585, Japan; Tel: +81-75-724-7781, Fax: +81-75-724-7799; E-mail: myamaguc@kit.ac.jp

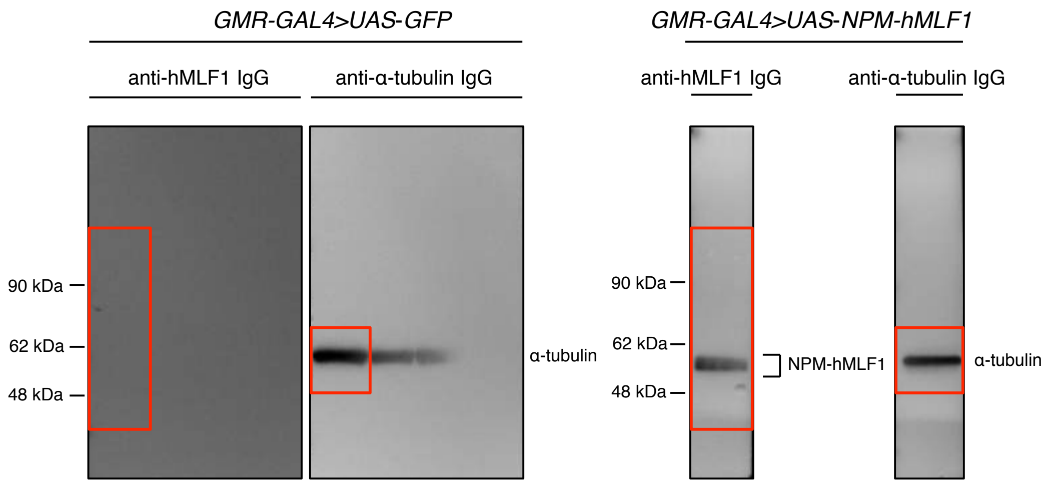

Figure S1. The full-length images of Fig. 3a. Red boxes represent the area of trimmed panels in Fig. 3a.

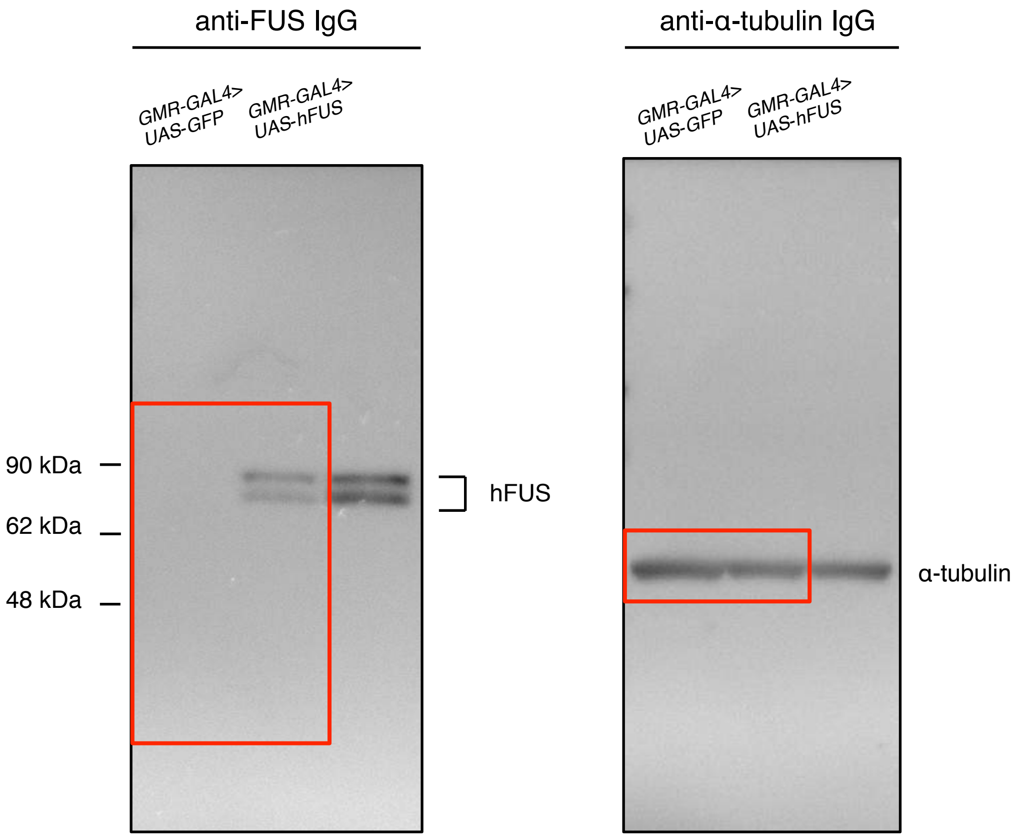

Figure S2. The full-length images of Fig. 4a. Red boxes represent the area of trimmed panels in Fig. 4a.

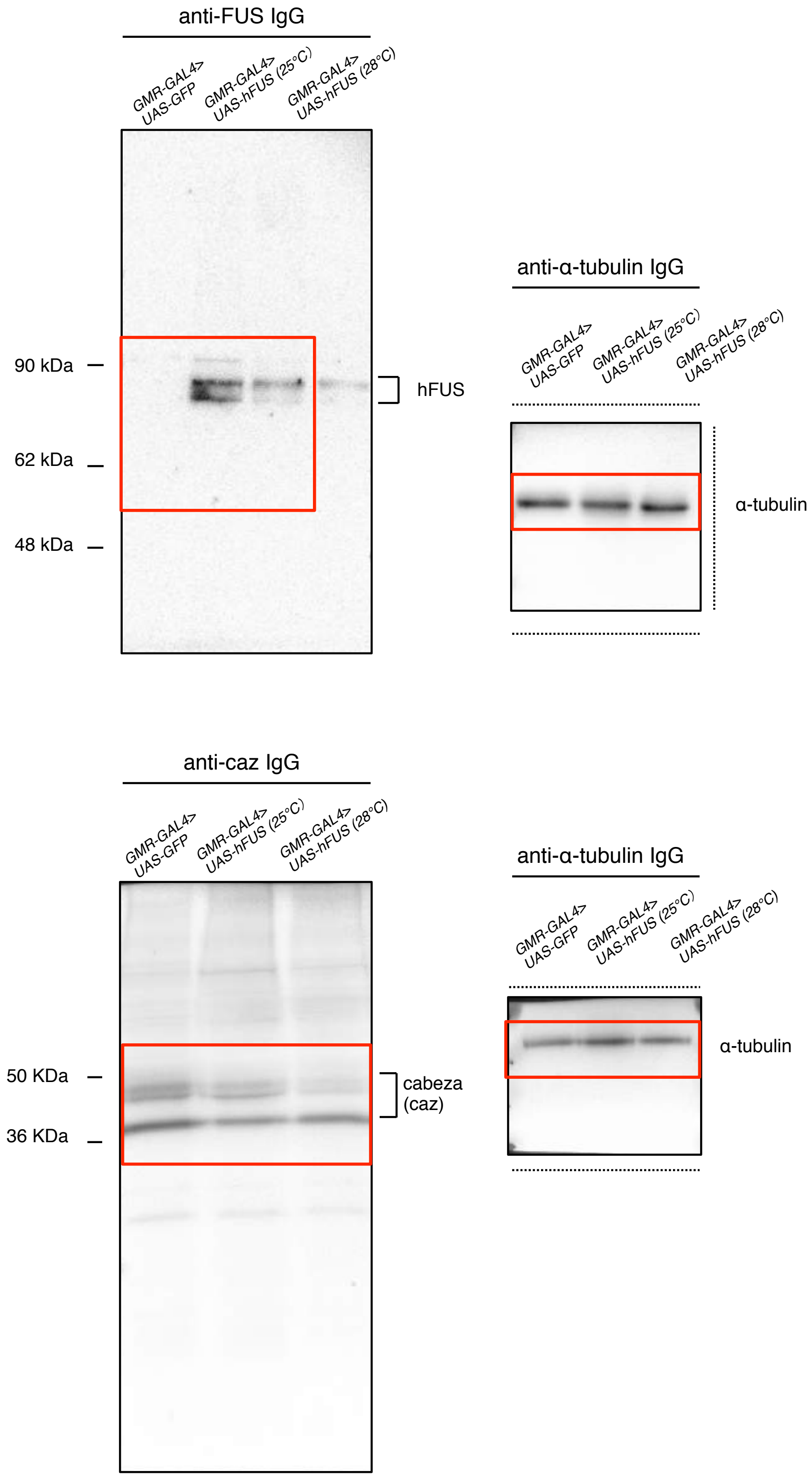

Figure S3. The full-length images of Fig. 5a. Red boxes represent the area of trimmed panels in Fig. 5a. Black dotted lines indicates the area to cut off.

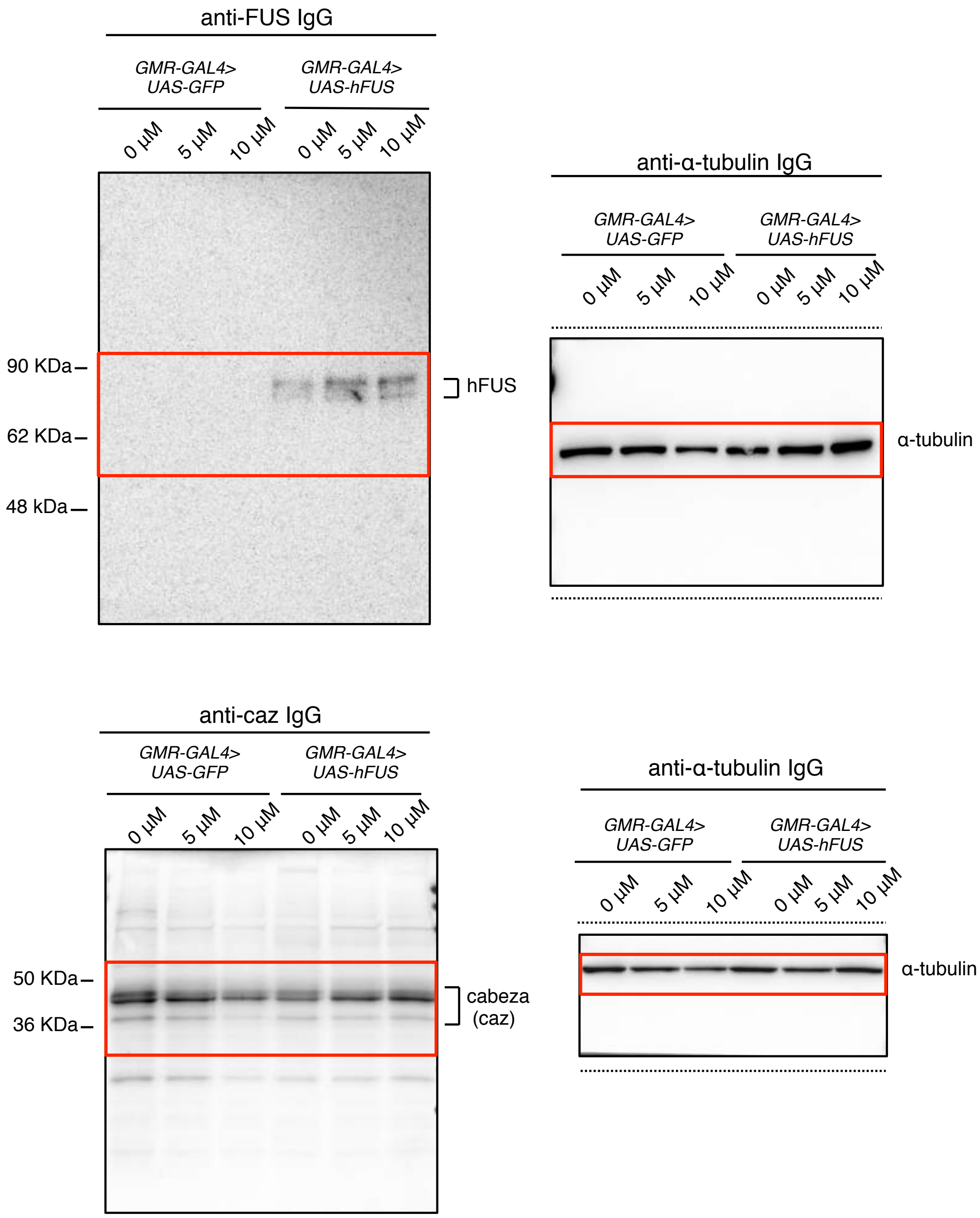

Figure S4. The full-length images of Fig. 5d. Red boxes represent the area of trimmed panels in Fig. 5d. Black dotted lines indicates the area to cut off.

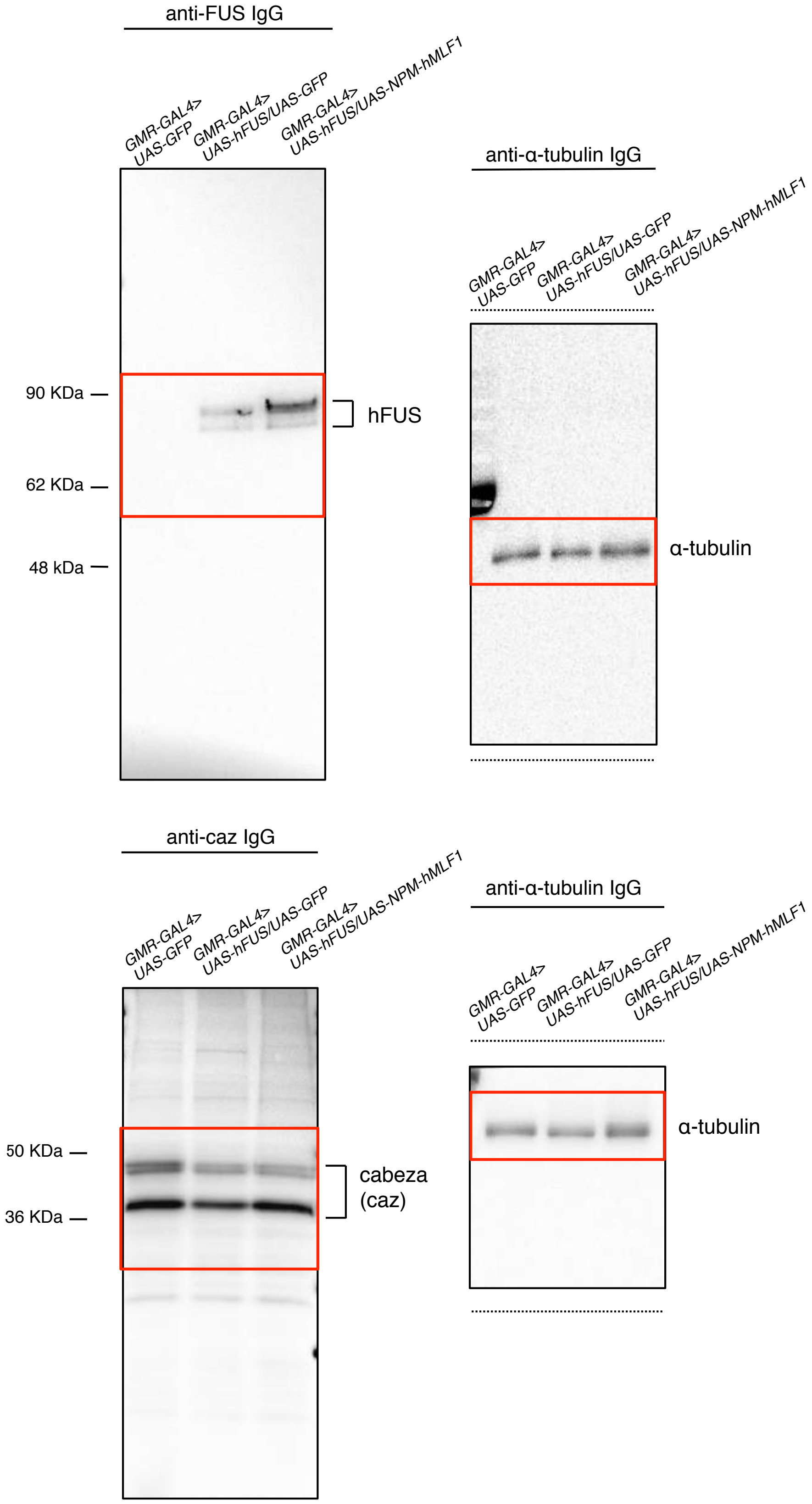

Figure S5. The full-length images of Fig. 6a. Red boxes represent the area of trimmed panels in Fig. 6a. Black dotted lines indicates the area to cut off.

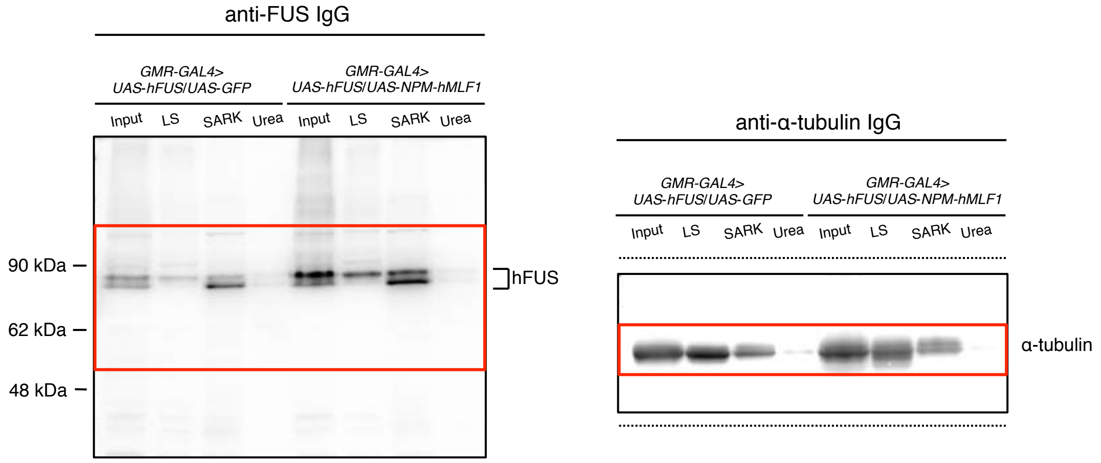

Figure S6. The full-length images of Fig. 7b. Red boxes represent the area of trimmed panels in Fig. 7b. Black dotted lines indicates the area to cut off.
